# Supplementary material for: Second-line treatment in advanced gastric cancer: Data from the Spanish AGAMENON registry
Source: PLoS One. 2020 Jul 31;15(7):e0235848. doi: 10.1371/journal.pone.0235848 (PMC7394396; doi:10.1371/journal.pone.0235848)
Supplement: S1 Table — (DOCX) [file pone.0235848.s001.docx]

|  | **Total, n (%),**  **1326 (100)** | **MonoCT, n (%), 755 (56.9)** | **Plat reint n (%),**  **110 (8.2)** | **Poly-CT, n (%),**  **199 (15.0)** | **Ram-CT, n (%),**  **167 (12.5)** | **Trastu + CT, n (%),**  **81 (6.1)** | **Ramu, n (%),**  **14 (1.0)** |
| --- | --- | --- | --- | --- | --- | --- | --- |
| Age, median (range) | 62 (20-86) | 63 (22-86) | 61 (34-86) | 59 (20-84) | 59 (20-81) | 63 (24-85) | 66 (46-81) |
| Sex, male | 965 (72.1) | 554 (73.4) | 81 (73.6) | 134 (67.3) | 114 (68.3) | 66 (81.5) | 7 (50) |
| Lauren, diffuse | 541 (40.8) | 298 (39.5) | 47 (42.7) | 95 (47.7) | 81 (48.5) | 13 (16.0) | 7 (50.0) |
| HER2, positive | 318 (24.0) | 163 (21.6) | 10 (9.1) | 22 (11.1) | 42 (25.1) | 81 (100) | 0 |
| PFS-1, median, 95% CI | 6.8 (6.5-7.1) | 6.4 (6.1-6.8) | 6.8 (5.8-9.5) | 6.8 (6.0-7.7) | 6.9 (6.0-7.6) | 9.7 (8.4-11.6) | 7.3 (4.7-12.5) |
| Best response to first line  *Complete*  *Partial*  *Stabilization*  *Progression* | 18 (1.4)  465 (35.1)  570 (43.0)  273 (20.6) | 9 (1.2)  253 (33.5)  325 (43.0)  168 (22.3) | 2 (1.8)  44 (40.0)  46 (41.8)  18 (16.4) | 1 (0.5)  69 (34.7)  78 (39.2)  51 (25.6) | 6 (3.6)  49 (29.3)  86 (51.5)  26 (15.6) | 0  47 (58.0)  26 (32.1)  8 (9.9) | 0  3 (21.4)  9 (64.3)  2 (14.3) |
| No PD to platin | 733 (55.3) | 397 (54.2) | 80 (10.9) | 107 (14.6) | 89 (12.1) | 54 (7.4) | 6 (0.8) |
| ECOG-PS, 0-1 | 1210 (91.3) | 682 (90.3) | 104 (94.5) | 183 (92.0) | 155 (92.8) | 75 (92.6) | 11 (78.6) |
| Burden of liver disease>50% | 247 (18.6) | 145 (19.2) | 18 (16.4) | 32 (16.1) | 25 (15) | 27 (33.3) | 0 |
| Number of metastases, >2 | 332 (25.9) | 190 (25.5) | 23 (20.9) | 48 (24.1) | 38 (22.8) | 31 (38.3) | 2 (14.3) |
| First-line CT  *Antracycline-based*  *Cisplatin-based*  *Docetaxel-based*  *Irinotecan-based*  *Other*  *Oxaliplatin-based* | 285 (21.5)  302 (22.8)  154 (11.6)  25 (1.9)  62 (4.7)  498 (37.6) | 145 (19.2)  206 (27.3)  70 (9.3)  15 (2.0)  39 (5.2)  280 (37.1) | 40 (36.3)  17 (15.5)  27 (24.5)  0  6 (5.5)  75 (37.7) | 43 (21.6)  22 (11.1)  46 (23.1)  5 (2.5)  8 (4.0)  75 (37.7) | 41 (24.6)  34 (20.4)  6 (3.6)  3 (1.8)  3 (1.8)  80 (47.9) | 12 (14.8)  23 (28.4)  5 (6.2)  1 (1.2)  6 (7.4)  34 (42) | 4 (28.6)  0  0  1 (7.1)  0  9 (64.3) |

**S1 Table.** Characteristics at the time of diagnosis by second-line strategy.

* The percentages refer to the columns
