# Supplementary material for: Second-line treatment in advanced gastric cancer: Data from the Spanish AGAMENON registry
Source: PLoS One. 2020 Jul 31;15(7):e0235848. doi: 10.1371/journal.pone.0235848 (PMC7394396; doi:10.1371/journal.pone.0235848)
Supplement: S2 Table — (DOCX) [file pone.0235848.s002.docx]

**S2 Table.** Chemotherapy regimens used based on HER2 status.

| **Variable** | **HER2-positive, n (%)**  **N=318** | **HER2-negative, n (%)**  **N=1008** |
| --- | --- | --- |
| Paclitaxel | 60 (18.9) | 224 (22.2) |
| Docetaxel | 43 (13.5) | 137 (13.6) |
| Paclitaxel-ramucirumab | 40 (12.6) | 125 (12.4) |
| Irinotecan | 37 (11.6) | 164 (16.3) |
| Paclitaxel-trastuzumab | 23 (7.2) | - |
| FOLFIRI | 20 (6.3) | 154 (15.3) |
| Other | 16 (5.0) | 25 (2.5) |
| Fluoropyrimidine-trastuzumab | 14 (4.4) | - |
| Irinotecan-trastuzumab | 9 (2.8) | - |
| XP-trastuzumab | 7 (2.2) | - |
| Docetaxel-trastuzumab | 7 (2.2) | - |
| CAPOX-trastuzumab | 6 (1.9) | - |
| CAPOX | 6 (1.9) | 65 (6.4) |
| Paclitaxel-ramucirumab-trastuzumab | 5 (1.6) | - |
| FOLFIRI-trastuzumab | 4 (1.3) | - |
| Fluoropyrimidine | 3 (0.9) | 33 (3.3) |
| Trastuzumab-other | 3 (0.9) | - |
| Irinotecan-docetaxel | 2 (0.6) | 15 (1.5) |
| Oxaliplatin | 2 (0.6) | 4 (0.4) |
| Cisplatin | 2 (0.6) | 3 (0.3) |
| Irinotecan-ramucirumab | 2 (0.6) | - |
| Trastuzumab monotherapy | 2 (0.6) | - |
| Oxaliplatin-other | 1 (0.3) | 14 (1.4) |
| XP | 1 (0.3) | 18 (1.8) |
| Oxaliplatin-docetaxel | 1 (0.3) | 3 (0.3) |
| Cisplatin-docetaxel-trastuzumab | 1 (0.3) | - |
| Cisplatin-docetaxel | **-** | 12 (1.2) |
| Ramucirumab | **-** | 14 (1.4) |
| Irinotecan-bevacizumab | - | 2 (0.2) |
| FOLFIRI-bevacizumab | - | 1 (0.1) |
