# Supplementary material for: Second-line treatment in advanced gastric cancer: Data from the Spanish AGAMENON registry
Source: PLoS One. 2020 Jul 31;15(7):e0235848. doi: 10.1371/journal.pone.0235848 (PMC7394396; doi:10.1371/journal.pone.0235848)
Supplement: S3 Table — (DOCX) [file pone.0235848.s003.docx]

**S3 Table.** Response rate depending on HER2 status and treatment strategy.

| **Variable** | **Complete response**  **%, 95% CI** | **Partial response**  **%, 95% CI** | **Stabilization**  **%, 95% CI** | **Response+ Stabilization,**  **%, 95% CI** | **Progression, %,**  **95% CI** | **Not available, %,**  **95% CI** |
| --- | --- | --- | --- | --- | --- | --- |
| **HER2-negative** |  |  |  |  |  |  |
| Ramucirumab | 0.0 (0.0-23.1) | 0.0 (0.0-23.1) | 28.5 (8.3-28.5) | 71.4 (41.8-91.6) | 0.0 (0.0-23.1) | 0.0 (0.0-23.1) |
| Mono-CT | 1.0 (0.3-2.1) | 8.4 (6.3-10.9) | 27.5 (23.9-31.3) | 36.9 (33.1-41.0) | 59.4 (55.3-63.4) | 3.5 (2.2-5.3) |
| Poly-CT | 0.5 (0.0-3.1) | 9.0 (5.2-14.2) | 34.5 (27.4-41.9) | 44.0 (36.6-51.7) | 55.3 (47.7-62.8) | 0.5 (0.0-3.1) |
| Ramucirumab-CT | 0.8 (0.0-4.3) | 12.8 (7.4-19.9) | 32.8 (26.6-41.7) | 46.4 (37.4-55.5) | 44.0 (35.1-53.1) | 9.6 (5.0-16.1) |
| Platinum reintroduction | 0.0 (0.0-3.6) | 16.0 (9.4-24.6) | 33.0 (23.9-43.1) | 49.0 (38.8-59.1) | 48.0 (37.9-58.2) | 3.0 (0.6-8.5) |
| **HER2-positive** |  |  |  |  |  |  |
| Mono-CT | 0.6 (0.0-3.3) | 11.0 (6.6-16.8) | 21.4 (15.4-28.5) | 33.1 (25.9-40.9) | 64.4 (56.5-71.7) | 2.4 (0.6-6.1) |
| Poly-CT | 0.0 (0.0-15.4) | 18.1 (5.1-40.2) | 22.7 (7.8-45.3) | 40.9 (20.7-63.6) | 54.5 (32.2-75.6) | 4.5 (0.1-22.8) |
| Ramucirumab-CT | 0.0 (0.0-8.4) | 30.9 (17.6-47.0) | 23.8 (12.0-39.4) | 54.7 (38.6-70.1) | 35.7 (21.5-51.9) | 9.5 (2.6-22.6) |
| CT- Trastuzumab | 0.0 (0.0-4.4) | 29.6 (19.9-40.8) | 27.1 (17.8-38.1) | 56.7 (45.3-67.7) | 39.5 (28.8-50.9) | 3.7 (0.7-10.4) |
| Platinum reintroduction | 0.0 (0.0-30.8) | 20.0 (2.5-55.6) | 40.0 (12.1-73.7) | 60.0 (26.2-87.8) | 40.0 (12.1-73.7) | 0.0 (0.0-30.8) |
