# Supplementary material for: Second-line treatment in advanced gastric cancer: Data from the Spanish AGAMENON registry
Source: PLoS One. 2020 Jul 31;15(7):e0235848. doi: 10.1371/journal.pone.0235848 (PMC7394396; doi:10.1371/journal.pone.0235848)
Supplement: S4 Table — (DOCX) [file pone.0235848.s004.docx]

**S4 Table.** Correlation between progression-free survival to first-line of treatment and overall survival.

| **Variable** | **Kendall’s τ** |
| --- | --- |
| Complete series | 0.613 |
| Subgroup receiving second-line | 0.539 |
| Second-line, HER2-positive | 0.566 |
| Second-line, HER2-negative | 0.525 |
| Second line, intestinal Lauren | 0.549 |
| Second line, diffuse Lauren | 0.530 |
| Second-line, ramucirumab + CT | 0.413 |
| Second-line, monochemotherapy | 0.539 |
| Second-line, polichemotherapy | 0.503 |
| Second-line, trastuzumab + chemoterapy | 0.566 |
| Second-line, platinum-reintroduction | 0.585 |

The estimates come from Clayton copula models using bivariate survival data.
