# Supplementary figures and images for: Second-line treatment in advanced gastric cancer: Data from the Spanish AGAMENON registry
Source: PLoS One. 2020 Jul 31;15(7):e0235848. doi: 10.1371/journal.pone.0235848 (PMC7394396; doi:10.1371/journal.pone.0235848)

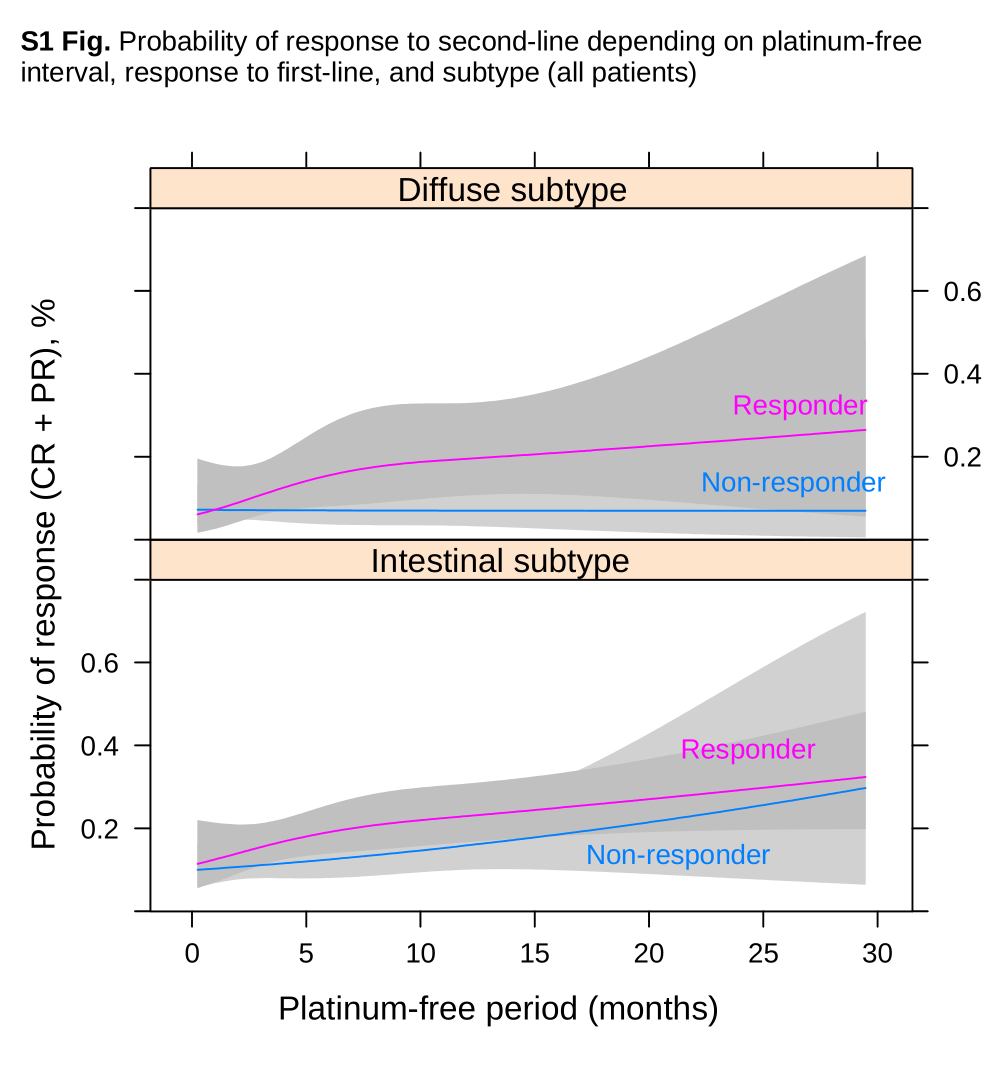

Supplement: S1 Fig — (TIFF) [file pone.0235848.s005.tiff]

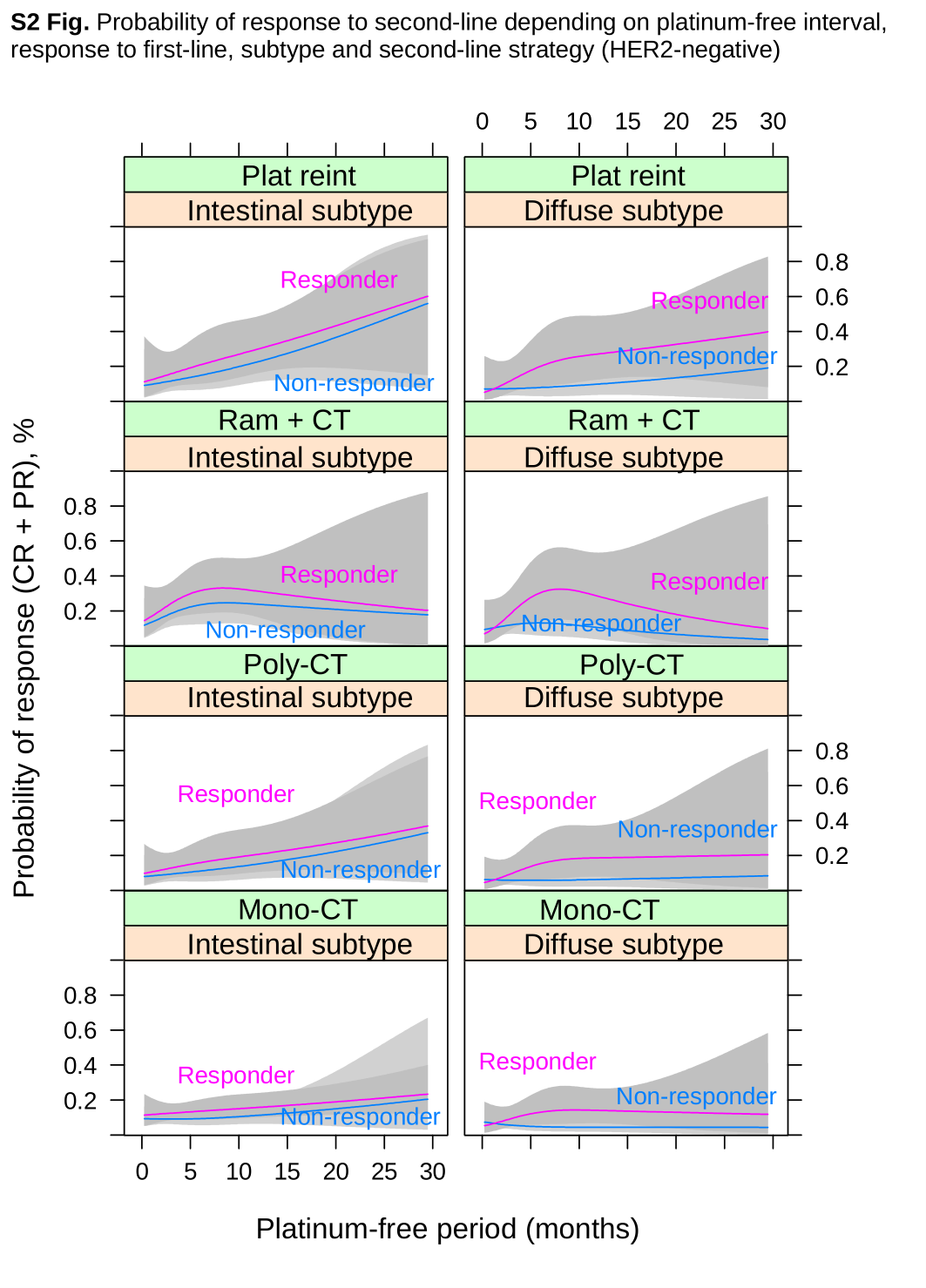

Supplement: S2 Fig — (TIFF) [file pone.0235848.s006.tiff]

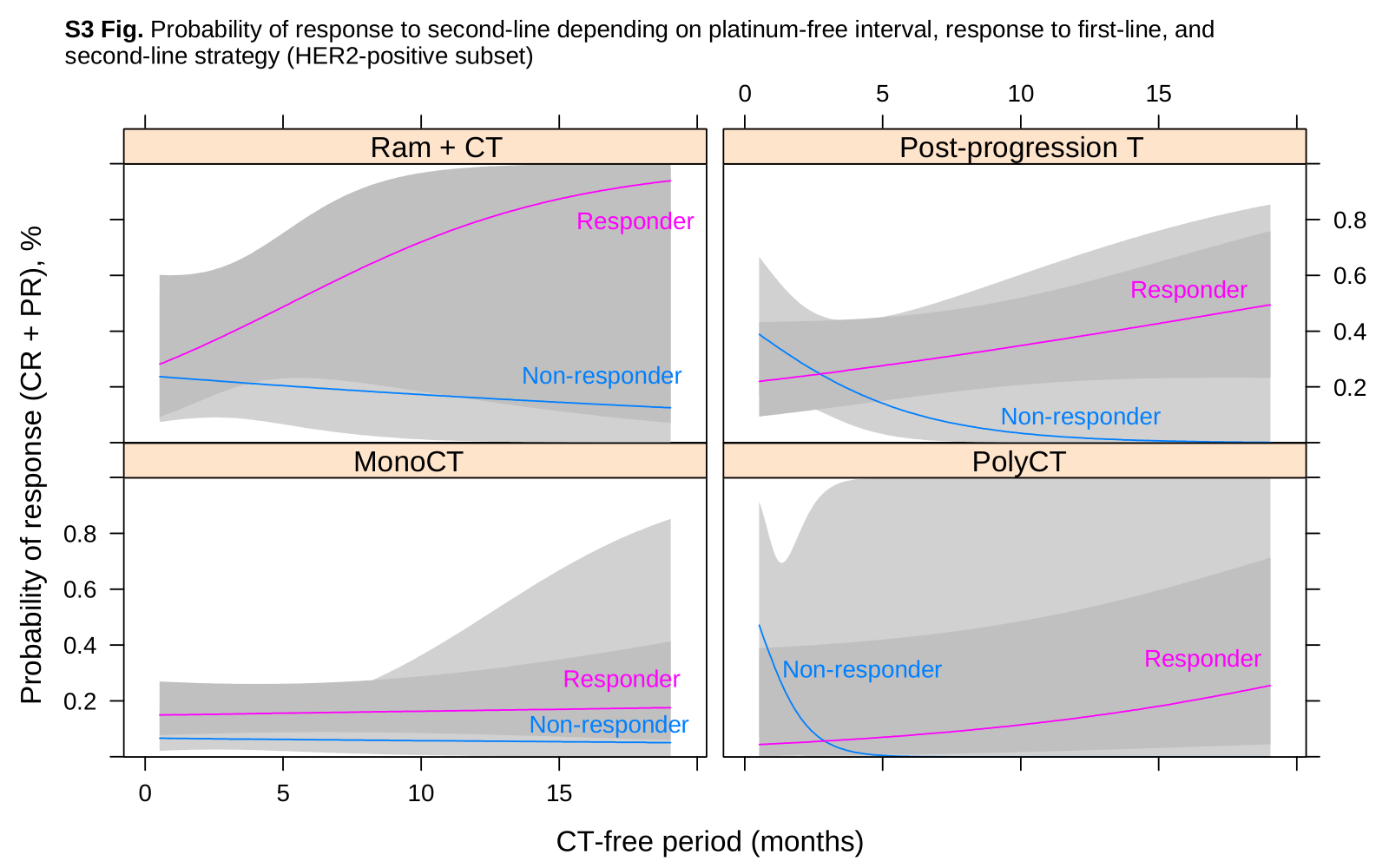

Supplement: S3 Fig — (TIFF) [file pone.0235848.s007.tiff]
