# Supplementary material for: Second-line treatment in advanced gastric cancer: Data from the Spanish AGAMENON registry
Source: PLoS One. 2020 Jul 31;15(7):e0235848. doi: 10.1371/journal.pone.0235848 (PMC7394396; doi:10.1371/journal.pone.0235848)
Supplement: Attachment — Submitted filename: Response to reviewers.docx [file pone.0235848.s008.docx]

**Response to reviewers**

**Responses are written in red.**

Dear editor,

In accordance with the Editor’s suggestions and concerns regarding the manuscript entitled, “Second-line treatment in advanced gastric cancer: data from the Spanish AGAMENON registry”, please find the new version enclosed.

On behalf of all the co-authors, I would like to thank the editor and the reviewers for their thoughtful and insightful comments on our work, which we have considered very closely, while preparing this revised version of the manuscript as detailed in the following point-by-point reply.

We trust that we have addressed all the issues raised by the reviewers to their satisfaction and you now find the manuscript suitable for publication in your journal.

Thank-you very much for your consideration.

Respectfully,

MD PhD, Javier Gallego Plaza

Additional Editor Comments:

In general the paper was well-written, a lot of efforts were spent on it. In addition to addressing the reviewers' concerns, you have to emphasize the main outstanding points of your paper that will catch the readers attention to your paper. Emphasize the points what this retrospective analysis adds or confirms to current knowledge. Consequently you should re-write the conclusion part of the paper since there is no take home points or important findings there. The last para of discussion in unnecessary please delete it (beginning with the readers must..). According to the heterogeneity of the treatment groups cox-regression analysis does not seem to be appropriate unless the including variables are appropriately described for this analysis, since there were lots variables (age, gender, tumor type, performance status, etc). Which model did you use for cox-regression?

The las part of the discussion has been deleted.

Conclusion has been re-written in order to stand out take home points and important findings.

We agree with the editor on this point, as it is important that the reader understands the specification of the multivariable model. We have therefore added the following paragraph in the statistics section:

*“No data-driven criteria were used for the model specification. The covariates for the multivariable model were chosen by theoretical considerations, as recommended in the literature [41]. Thus, ECOG PS (<2, ≥2), Lauren's histopathological subtype (intestinal, diffuse), number of metastatic sites (≤2, >2), liver tumor burden (≤50, >50%), HER2 status (negative, positive), PFS-1, and best response to first-line (complete or partial response, stable disease, progressive disease) were used as confounding factors. Restricted cubic splines were used to model the non-linear effect of PFS-1.”*

*A new reference has been included and previous numbers have been modified accordingly:*

41. Harrell F. Regression modeling strategies: with applications to linear models, logistic and ordinal regression, and survival analysis. 2nd ed. New York: Springer; 2015.

Journal Requirements:

When submitting your revision, we need you to address these additional requirements.

1. Please ensure that your manuscript meets PLOS ONE's style requirements, including those for file naming. The PLOS ONE style templates can be found at

[https://journals.plos.org/plosone/s/file?id=wjVg/PLOSOne_formatting_sample_main_body.pdf](https://journals.plos.org/plosone/s/file?id=wjVg/PLOSOne_formatting_sample_main_body.pdf) and

[https://journals.plos.org/plosone/s/file?id=ba62/PLOSOne_formatting_sample_title_authors_affiliations.pdf](https://journals.plos.org/plosone/s/file?id=ba62/PLOSOne_formatting_sample_title_authors_affiliations.pdf)

Text has been reviewed in order to meet PLOS ONE’s style requirements.

References have been reviewed and adapted to requirements.

2. Please include your tables as part of your main manuscript and remove the individual files. Please note that supplementary tables should remain as separate "supporting information" files.

Tables and figures have been included as part of the main manuscript.

Supplementary tables and figures remain as separate “supporting information” files.

3. Please provide additional details regarding participant consent.

In the ethics statement in the Methods and online submission information, please ensure that you have specified whether participants who were not alive at data collection had previously provided written informed consent to use their medical records for the purposes of research.

Additional information has been specified in Material and methods: Patients and design.

4. Thank you for including your ethics statement:

'A multicenter Research Ethics Committee from all the Autonomous Communities and participating hospitals approved the study.'

(a) Please amend your current ethics statement to include the full name of the ethics committee/institutional review board(s) that specifically approved your specific study.

The name of the ethics committee/institutional review board who first approved the specific study, which was adressed as reference committee, is stated in the text (Material and methods: Patients and design).

(b) Once you have amended this/these statement(s) in the Methods section of the manuscript, please add the same text to the “Ethics Statement” field of the submission form (via “Edit Submission”).

Modified version of the “Ethics Statement”, with already mentioned changes, has been included in the submission form.

For additional information about PLOS ONE ethical requirements for human subjects research, please refer to [http://journals.plos.org/plosone/s/submission-guidelines#loc-human-subjects-research](http://journals.plos.org/plosone/s/submission-guidelines#loc-human-subjects-research).

5. Thank you for providing the following Funding Statement:

"JG Phd. Declares advisory role for Amgen, Bayer, BMS, Ipsen, Lilly, Merck, Roche, Servier. Travel grants from Novartis, Amgen.
None of the funders played any role in the study design, data collection and analysis, decision to publish, or preparation of the manuscript."

We note that one or more of the authors is affiliated with the funding organization, indicating the funder may have had some role in the design, data collection, analysis or preparation of your manuscript for publication; in other words, the funder played an indirect role through the participation of the co-authors.

a. If the funding organization did not play a role in the study design, data collection and analysis, decision to publish, or preparation of the manuscript and only provided financial support in the form of authors' salaries and/or research materials, please review your statements relating to the author contributions, and ensure you have specifically and accurately indicated the role(s) that these authors had in your study in the Author Contributions section of the online submission form. Please make any necessary amendments directly within this section of the online submission form.  Please also update your Funding Statement to include the following statement: “The funder provided support in the form of salaries for authors [insert relevant initials], but did not have any additional role in the study design, data collection and analysis, decision to publish, or preparation of the manuscript. The specific roles of these authors are articulated in the ‘author contributions’ section.”

If the funding organization did have an additional role, please state and explain that role within your Funding Statement.

The AGAMENON group neither received funding or financial support nor payed salaries to none of the authors for study design, data collection, decision to publish, authors contributions, and preparation of the manuscript for this study.

This sentence has been added to the Funding statement

b. Please also provide an updated Competing Interests Statement declaring this commercial affiliation along with any other relevant declarations relating to employment, consultancy, patents, products in development, or marketed products, etc.

Within your Competing Interests Statement, please confirm that this commercial affiliation does not alter your adherence to all PLOS ONE policies on sharing data and materials by including the following statement: "This does not alter our adherence to  PLOS ONE policies on sharing data and materials.” (as detailed online in our guide for authors http://journals.plos.org/plosone/s/competing-interests) . If this adherence statement is not accurate and  there are restrictions on sharing of data and/or materials, please state these. Please note that we cannot proceed with consideration of your article until this information has been declared.

Detailed adherence to PLOS ONE policies on sharing data and materials has been specified in Competing Interests Statement.

Please know it is PLOS ONE policy for corresponding authors to declare, on behalf of all authors, all potential competing interests for the purposes of transparency. PLOS defines a competing interest as anything that interferes with, or could reasonably be perceived as interfering with, the full and objective presentation, peer review, editorial decision-making, or publication of research or non-research articles submitted to one of the journals. Competing interests can be financial or non-financial, professional, or personal. Competing interests can arise in relationship to an organization or another person. Please follow this link to our website for more details on competing interests: http://journals.plos.org/plosone/s/competing-interests

6. Please amend your manuscript to include your abstract after the title page.

Abstract has been included after the tittle page.

7. Please upload a copy of Figure 4, to which you refer in your text on page 17. If the figure is no longer to be included as part of the submission please remove all reference to it within the text.

Figure 4 is included as well as all other tables and figures (except supplementary) in the main manuscript, as requested in point 2 of this text.

Moreover, all figures (main manuscript and supplementary) are included also as separate files in the submission.

8. Please include captions for your Supporting Information files at the end of your manuscript, and update any in-text citations to match accordingly. Please see our Supporting Information guidelines for more information: http://journals.plos.org/plosone/s/supporting-information

Captions for Supporting Information files have been included at the end of the manuscript.

Text has been updated in order to match accordingly with captions.

9. Your ethics statement must appear in the Methods section of your manuscript. If your ethics statement is written in any section besides the Methods, please move it to the Methods section and delete it from any other section. Please also ensure that your ethics statement is included in your manuscript, as the ethics section of your online submission will not be published alongside your manuscript.

The ethics statement has been included in the Methods section of the manuscript.

The ethics statement has been removed from the Disclaimer section at the end of the manuscript.

[Note: HTML markup is below. Please do not edit.]

Reviewers' comments:

Reviewer's Responses to Questions

**Comments to the Author**

1. Is the manuscript technically sound, and do the data support the conclusions?

The manuscript must describe a technically sound piece of scientific research with data that supports the conclusions. Experiments must have been conducted rigorously, with appropriate controls, replication, and sample sizes. The conclusions must be drawn appropriately based on the data presented. 

Reviewer #1: Yes

Reviewer #2: Partly

No comment.

2. Has the statistical analysis been performed appropriately and rigorously? 

Reviewer #1: Yes

Reviewer #2: No

The statistical plan of this study was designed according to the descriptive objective of the study, using classic, widely used methods. As described in methods, survival was evaluated using the non-parametric Kaplan-Meier estimator, and multivariable Cox regression, largely meeting the assumptions of these models. Quantification between PFS & OS has also been performed by a classical approach. These methods were implemented using statistical software widely used by academics around the world (R with its basic survival libraries). The results have also been honestly reported. We therefore consider that the handling of the data has been optimal and robust.

3. Have the authors made all data underlying the findings in their manuscript fully available?

The [PLOS Data policy](http://www.plosone.org/static/policies.action#sharing) requires authors to make all data underlying the findings described in their manuscript fully available without restriction, with rare exception (please refer to the Data Availability Statement in the manuscript PDF file). The data should be provided as part of the manuscript or its supporting information, or deposited to a public repository. For example, in addition to summary statistics, the data points behind means, medians and variance measures should be available. If there are restrictions on publicly sharing data—e.g. participant privacy or use of data from a third party—those must be specified.

Reviewer #1: Yes

Reviewer #2: Yes

 No comment.

4. Is the manuscript presented in an intelligible fashion and written in standard English?

PLOS ONE does not copyedit accepted manuscripts, so the language in submitted articles must be clear, correct, and unambiguous. Any typographical or grammatical errors should be corrected at revision, so please note any specific errors here.

Reviewer #1: Yes

Reviewer #2: Yes

 No comment.

5. Review Comments to the Author

Please use the space provided to explain your answers to the questions above. You may also include additional comments for the author, including concerns about dual publication, research ethics, or publication ethics. (Please upload your review as an attachment if it exceeds 20,000 characters)

Reviewer #1: Author describe and comparison the CTs for 2nd line GC patients from real-world data of Spain.

1. In multivariate analysis for survival, did you include the potential prognostic factor, for example PS, ALP, No of mets and others? As you mentioned, the patients who received mono CT as second line have different condition compared to polyCT.

We agree with the reviewer that this point merits clarification. Several covariates were used in the multivariable analysis to reduce confounding bias, including ECOG PS, Lauren's histopathological subtype, number of metastatic sites, liver tumor burden, HER2 status, PFS-1, and best response to first-line. We have added a paragraph to better explain the specification of the multivariable model:

*“No data-driven criteria were used for the model specification. The covariates for the multivariable model were chosen by theoretical considerations, as recommended in the literature [1]. Thus, ECOG PS (<2, ≥2), Lauren's histopathological subtype (intestinal, diffuse), number of metastatic sites (≤2, >2), liver tumor burden (≤50, >50%), HER2 status (negative, positive), PFS-1, and best response to first-line (complete or partial response, stable disease, progressive disease) were used as confounding factors. Restricted cubic splines were used to model the non-linear effect of PFS-1.”*

*A new reference has been included and previous numbers have been modified accordingly:*

41. Harrell F. Regression modeling strategies: with applications to linear models, logistic and ordinal regression, and survival analysis. 2nd ed. New York: Springer; 2015.

2. Do you have a data of the patients who received ICI as 3rd and later line? Because the response of ICI may continue for a long time, the use of ICI may influence the survival of each groups.

We appreciate your pertinent comment.

As this manuscript shows, survival time after first-line progression is becoming responsblee for a larger portion of overall time survival than previously; hence correlation of first-line progression free survival and overall survival is decreasing.

Second line treatment options with proven efficacy and the progressive introduction and use of further lines of treatment, along with increasing immunotherapy use, are very important causes of this finding. This is the reason why we are also very interested in analysing patterns of use and results of further lines of treatment in advanced gastric cancer.

The first version of the AGAMENON National Registry for advanced gastric cancer focused mainly on the epidemiological and diagnostic data, as well as the patterns and results of first-line and second-line treatment, which were covered in depth. Unfortunately, we did not cover treatment beyond the second line at that time in our registry.

In May 2020 the amended version of the Spanish National AGAMENON Registry protocol was approved. One of the most important changes in this amended version is the quantity of information captured in detail related to second and further lines of treatment.

We hope we will be able to increase our knowledge and understanding of the patterns of use and results of third and further lines of treatment, as well as of immunotherapy, in advanced gastric cancer with future planned analysis of our Spanish National AGAMENON Registry.

3. In Asian countries, first line platinum agent continue more than 6 courses if the patients have no toxicities and inconvenient. The re-introduction of platinum depends of the strategy of using of first line platinum administration. How the first line platinum are used for the patients with GC in Spain?

We would like to thank the reviewer for his pertinent appreciation which we explain below.

No formal study has specifically addressed the duration of first-line treatment in advanced gastric cancer. First-line treatment randomized clinical trials have had different approaches to treatment duration, from limited to a certain number of cycles to those that stablished treatment to progression or unacceptable toxicity. Limiting the number of cycles of chemotherapy has been an increasing strategy with the introduction of targeted therapies and immunotherapy, as both are also used as maintenance treatments. Limited duration of first-line treatment is now also considered in order to preserve functional status and avoid cumulative toxicity, once there are treatment options with proven efficacy in further lines.

We have already explored the duration of first-line treatment in the Spanish National AGAMENON Registry (Viúdez A, Carmona-Bayonas A, Gallego J, et al. Optimal duration of first-line chemotherapy for advanced gastric cancer: data from the AGAMENON registry. Clin Transl Oncol 2019 - <https://doi.org/10.1007/s12094-019-02183-y>). Our analysis based on 415 patients with no progression on 6-month CT showed: 18% of patients maintained full treatment until progression, 30% of patients discontinued platinum and maintained fluoropyrimidine until progression, and 52% of patients completely withdrew treatment prior to progression. This finding, along with the evidence and recommendations now included in guidelines, makes the option of limited duration of first-line treatment something to consider in real-world evidence.

Reviewer #2: Sanchis et al presented a retrospective/observational study on second line treatment options for advanced gastric cancer AGC according to clinical practice in several cancer centers in Spain and Chile. AGC, after first line therapy failure, has poor prognosis but recent developments has added to the clinical practice scenario, new therapeutic approaches with a clear benefit on survival. The addition of VEGFR2 antagonist ramucirumab to taxanes, for instance, increased OS compared to mono-chemotherapy regimens.
The topic addressed by this manuscript is important and the paper is well written, however it lacks novelty and simply provide a description of several regimens used in second-line setting throughout the last 12 years.

The study performed based on the Spanish National AGAMENON Registry has analized the pattern of use and results of second-line treatment and is the largest published so far. It not only describes but also evaluates treatment outcomes treatment trends in time, new treatment options incorporation; and the evolution of the correlation between first-line progression free survival and overall survival.

It appears clear that the use of ramicurumab is climbing in the most recent time due to the wide spreading of this agents from 2015 when RAINBOW trial was published. Moreover, the methods appear to be sound but the correlation of PFS and OS with each treatment strategy in the results section is not easily readable and should be expanded and better performed to ensure that readers understand exactly what the researchers wanted to state.

As a clarification to your request, a comment has been added to the description of the correlation of PFS and OS in the results section to make them more understandable.

Thirdly, platinum re-introduction, when feasible, could be a valid therapeutic option, however the conclusion of the manuscript appears to be way stronger due to low number (8.3%), the paucity of patients that will be able to tolerate the reintroduction of platinum-doublet in this setting and the results, totally comparable to Ram-taxane, which shows a better toxicity profile.

Minor changes have been made to the conclusion of the manuscript in order to highlight the option and efficacy of Ram-taxane and to restrict the option of rechallenge.

Lastly, the dissertation on PFS as adequate surrogate of OS is interesting. This is a current unmet need in oncology research. Specifically, in this study, authors focused on PPS (post progression survival). According to their data, it that could have some relevance in Her2 positive AGC since the anti-her2 action of trastuzumab, even though a radiologic progression, keeps the ability to control the Her-2 enriched population which could lead to a longer overall survival. However, for Her2-negative tumors, the magnitude of the benefit is lower, reaching less than 3 months in the best subgroup. In this case, QoL has much more impact and relevance for patients and should be primarily assessed in studies on poor prognosis cancer after first setting.

We totally agree with this comment.

We think this is the reason why recent randomized clinical trials in further lines of gastric cancer treatment have incorporated QoL analysis.

We have also modified our Spanish National AGAMENON Registry in order to include detailed information on toxicity related to further lines of treatment in real-world evidence, as we believe this may be valuable information when considering to choose between different treatment options.

6. PLOS authors have the option to publish the peer review history of their article ([what does this mean?](https://journals.plos.org/plosone/s/editorial-and-peer-review-process#loc-peer-review-history)). If published, this will include your full peer review and any attached files.

If you choose “no”, your identity will remain anonymous but your review may still be made public.

**Do you want your identity to be public for this peer review?** For information about this choice, including consent withdrawal, please see our [Privacy Policy](https://www.plos.org/privacy-policy).

Reviewer #1: Yes: Ken Kato

Reviewer #2: No

No comment.

[NOTE: If reviewer comments were submitted as an attachment file, they will be attached to this email and accessible via the submission site. Please log into your account, locate the manuscript record, and check for the action link "View Attachments". If this link does not appear, there are no attachment files.]

While revising your submission, please upload your figure files to the Preflight Analysis and Conversion Engine (PACE) digital diagnostic tool, [https://pacev2.apexcovantage.com/](https://pacev2.apexcovantage.com/). PACE helps ensure that figures meet PLOS requirements. To use PACE, you must first register as a user. Registration is free. Then, login and navigate to the UPLOAD tab, where you will find detailed instructions on how to use the tool. If you encounter any issues or have any questions when using PACE, please email PLOS at [figures@plos.org](mailto:figures@plos.org). Please note that Supporting Information files do not need this step.
